# Developing a Novel Measure of Body Satisfaction Using Virtual Reality

**DOI:** 10.1371/journal.pone.0140158

**Published:** 2015-10-15

**Authors:** Clare K. Purvis, Megan Jones, Jakki O. Bailey, Jeremy Bailenson, C. Barr Taylor

**Affiliations:** 1 PGSP-Stanford Consortium, Department of Psychiatry & Behavioral Sciences, Stanford University School of Medicine, Stanford, California, United States of America; 2 Department of Psychiatry & Behavioral Sciences, Stanford University School of Medicine, Stanford, California, United States of America; 3 Department of Communication, Stanford University, Stanford, California, United States of America; Swansea University, UNITED KINGDOM

## Abstract

Body image disturbance (BID), considered a key feature in eating disorders, is a pervasive issue among young women. Accurate assessment of BID is critical, but the field is currently limited to self-report assessment methods. In the present study, we build upon existing research, and explore the utility of virtual reality (VR) to elicit and detect changes in BID across various immersive virtual environments. College-aged women with elevated weight and shape concerns (*n* = 38) and a non-weight and shape concerned control group (*n* = 40) were randomly exposed to four distinct virtual environments with high or low levels of body salience and social presence (i.e., presence of virtual others). Participants interacted with avatars of thin, normal weight, and overweight body size (BMI of approximately 18, 22, and 27 respectively) in virtual social settings (i.e., beach, party). We measured state-level body satisfaction (state BD) immediately after exposure to each environment. In addition, we measured participants’ minimum interpersonal distance, visual attention, and approach preference toward avatars of each size. Women with higher baseline BID reported significantly higher state BD in all settings compared to controls. Both groups reported significantly higher state BD in a beach with avatars as compared to other environments. In addition, women with elevated BID approached closer to normal weight avatars and looked longer at thin avatars compared to women in the control group. Our findings indicate that VR may serve as a novel tool for measuring state-level BID, with applications for measuring treatment outcomes. Implications for future research and clinical interventions are discussed.

## Introduction

Body image disturbance (BID) refers to negative self-evaluations of one’s physical appearance which lead to significant distress or impairment [1]. BID is a highly prevalent issue in the general population, particularly among young women [2,3]. BID is associated with depression and anxiety [4,5], impairment in social relationships [6], and perhaps most importantly, it is linked to the onset and maintenance of eating disorders (ED) [7–9].

While BID has been studied widely, certain important features of this phenomenon remain poorly understood. This is partly the result of existing assessment techniques, which limit the scope of BID research by focusing on BID main as a stable attitude, or trait; few measures assess the degree to which one’s body image fluctuates at a given moment in time [10]. Such momentary fluctuations in BID are referred to as one’s “state” body (dis)satisfaction (state BD); variability in state BD is an important predictor of the likelihood of developing ED, and an indicator of BID severity [11]. Despite its importance, factors that influence state BD are poorly understood, partly because state BD assessments rely on retrospective accounts or lack ecological validity [12].

Recent research suggests that automatic cognitive and behavioral processes are highly involved in the state BD; however, traditional self-report measures are unable to assess automatic processes because they originate outside of conscious awareness [12]. For example, despite having a worsening effect on state BD, women with BID, compared to those without BID, look longer at thin, appearance-ideal conforming female bodies [13], and are predisposed to overestimate the prevalence of negative social feedback about their own bodies [14]. Such processing errors are believed to reinforce negative body-related self-evaluations and, as a result, may cause or maintain disordered behaviors [12,15,16]. This has not been studied directly, as tools for measuring automatic processes and implicit behaviors in BID are nascent. Researchers have developed computer-based techniques to explore visual attention [17,18], but the methods have limited ecological validity. Understanding how automatic processes in BID function in real-world contexts, and how they relate to overt behavior is of vital importance in properly conceptualizing BID and in developing effective interventions.

Virtual reality (VR) offers tools to address limitations in BID assessment. VR has gained popularity in psychological research because it provides researchers excellent environmental control, high realism, and unparalleled control over assessment timing [19]. VR is especially well suited tool to study automatic non-verbal behaviors in realistic contexts, and behavior performed in VR has been shown to closely mirror real-world behaviors [20]. Further, VR enables researchers to measure behavior automatically and fairly unobtrusively, and attitudinal measures can be completed immediately following or even during experimental manipulations. By using VR, researchers can directly measure the relationships between explicit attitudes and behaviors, typically impossible with self-report questionnaires alone [21]. These advantages listed make VR an excellent tool for exploring emerging models of BID.

VR has been applied to BID assessment and intervention in past studies, and the present research aims to build on prior work in several important ways. First, VR studies have shown that individuals with ED exhibit strong affect responses to disorder-relevant virtual environments [22], but existing paradigms emphasize food and eating, and thus are not directly relevant to BID in the absence of eating pathology. Second, the majority of studies using VR to study BID have selected mood, anxiety, and perceptual body image distortions as primary outcome variables instead of measuring body image attitudes directly [19, 20]. Little is known about the relationship between environmental cues and state BD, although there is some evidence to suggest that social environments wherein the body is highly salient–such as a virtual swimming pool–may elicit changes in state BD among non-ED women [18]. Most importantly, no existing studies have explored behaviors associated with BID. We argue that VR offers vast opportunities to expand the field’s understanding of BID, particularly in terms of automatic behaviors, which may play an important role in maintaining disturbed body image.

### The Present Study

The present study presents a novel paradigm for measuring BID in VR. First, we propose to validate a series of virtual environments that are relevant to BID among women without eating pathology. The environments used in existing VR exposure protocols for ED patients were developed by administering traditional measures in the context of a virtual environment. We propose to use a similar method in which we determine the extent to which one’s experience in VR mirrors that expected in similar real–world situations. Observational, theoretical, and experimental studies suggest that situations in which one’s body is highly salient and/or in which one engages in body-based social comparison are highly relevant to BID [11]. We intend to manipulate these factors in VR to measure their influence on self-reported state BD.

Second, we aim to investigate the extent to which automatic nonverbal social behaviors are related to BID. VR allows us to explore visual attention in a realistic setting, whereas prior studies have been conducted in highly controlled laboratory settings by measuring attention toward static images, often presented on a computer screen. Visual gaze is easily measured in VR by deriving a user’s field of view as a function of her head movements (Bailenson et al., 2002). By measuring visual attention in virtual social contexts, we can determine whether attentional biases demonstrated in laboratory studies persist in situations in naturalistic settings, in which many stimuli (in addition to body-related cues) compete for one’s attention.

We further aim to extend previous work in automatic processes related to BID by exploring interpersonal approach behavior, which we believe may relate to BID. Specifically, previous VR studies in a variety of disciplines have shown interpersonal distance to be a sensitive indicator of implicit attitudes and evaluations [23]. Interpersonal distance has been studied for many years as an unconscious indicator of social attitudes for many years; early studies indicated that people consistently approach more closely to positively-evaluated others, whereas they maintain greater distance from others who are members of socially derogated groups [older distance studies]. In the VR literature, interpersonal distance refers to the space a participant places between him or herself and a digital human, called an avatar. Interpretations of interpersonal distance differ depending upon the context, but it is generally regarded as an indicator of global attitudes: standing closer to another person is perceived as a positive overall appraisal, whereas standing further away indicates dislike of the target or discomfort. Further, interpersonal distance is primarily automatic. Although, interpersonal distance has never been explored as an indicator of body image attitudes we expect it is related to automatic attention biases in BID. Determining the extent to which interpersonal behavior–both distance and visual gaze–are associated with BID in realistic settings may help to identify automatic systems that underlie BID, allowing for the development of more robust interventions.

Hypothesis I: Participants with greater weight-and-shape concerns will report higher levels of state BD across all environments compared to participants with low levels of weight-and-shape concerns.

Hypothesis II: Participants will report the most state BD in a VR environment with high body salience and social presence, compared to other environmental conditions.

Hypothesis III: Participants will vary their behavior toward female avatars based on their pre-existing levels of weight-and-shape concerns. Specifically, participants with elevated weight-and-shape concerns will approach more closely and demonstrate more visual attention toward thin avatars as compared to participants without elevated weight-and-shape concerns.

## Method

### Participants

A convenience sample was recruited using the student research participant pool of the Stanford University Communication Department during the 2012–2013 Winter and Spring quarters and the 2013–2014 Fall quarter. Students received course credit for participation in the study. All students in available courses completed an online prescreen questionnaire at the beginning of the quarter to determine their eligibility. The Weight Concerns Scale (WCS) was included in the online questionnaire to assess level of body image disturbance. WCS scores greater than 47 are associated with greater BID overall, and have been used to indicate increased risk for developing an eating disorder [24,25]. Students eligible for this study were randomly invited to participate based on their WCS score. Invitations to participate in the study were extended according to departmental policies to approximately equal numbers of female students with WCS total scores above and below 47, respectively.

A total of 112 students responded to an invitation to participate in the study. Of those 112 students, 103 students provided written informed consent and completed baseline assessments. Of the 103 students who completed baseline assessments, 24 were excluded from final analysis for the following reasons: (a) failure to meet inclusion criteria (*n* = 3); (b) problems with the recording and/or tracking equipment in the VR system (*n* = 14); and (c) onset of dizziness during VR exposure (*n* = 7). Seventy-nine students completed all phases of the VR exposure and post-test assessments. Of these, two participants were found to have partial data on baseline assessments and were excluded from analysis. Analyses were therefore conducted on a final sample of N = 77.

At baseline, participants were screened and sorted into two groups on the basis of WCS total scores: elevated overall BID (referred to as the “weight-concerned” group; *n* = 39), WCS > = 47 or controls (*n* = 40), WCS < 47. Participants who endorsed eating-related pathology consistent with the DSM-IV-TR eating disorder criteria were not included in the study and were provided with referral information for in-person and web-based mental health services on campus.

### Ethics Statement

All procedures and materials were approved by the Ethical Committee of the Institutional Review Board at Stanford University. Written consent was obtained from all participants; the individual pictured in this manuscript, who was not a study participant, has given written informed consent (as outlined in PLOS consent form) for his image to be used.

### Procedures

The lead researcher and one undergraduate research assistant were present for each trial. Upon arriving at the laboratory, all participants signed a consent form. Participants completed computer-based questionnaires assessing baseline weight-and-shape concerns, eating pathology, body satisfaction, and physical appearance comparison.

The researcher provided all participants with a verbal description of the purpose of the study explaining the components of the VR experience. Participants were told that they would be participating in a study examining whether certain virtual environments influenced women’s awareness of their own bodies and their behavior towards others. The researcher then assisted each participant in wearing the virtual reality headset. After completing the VR task, all participants received debriefing materials containing information about online resources for body image improvement and contact information for university counseling and psychological services.

#### Virtual Reality Task

**Fig 1:**
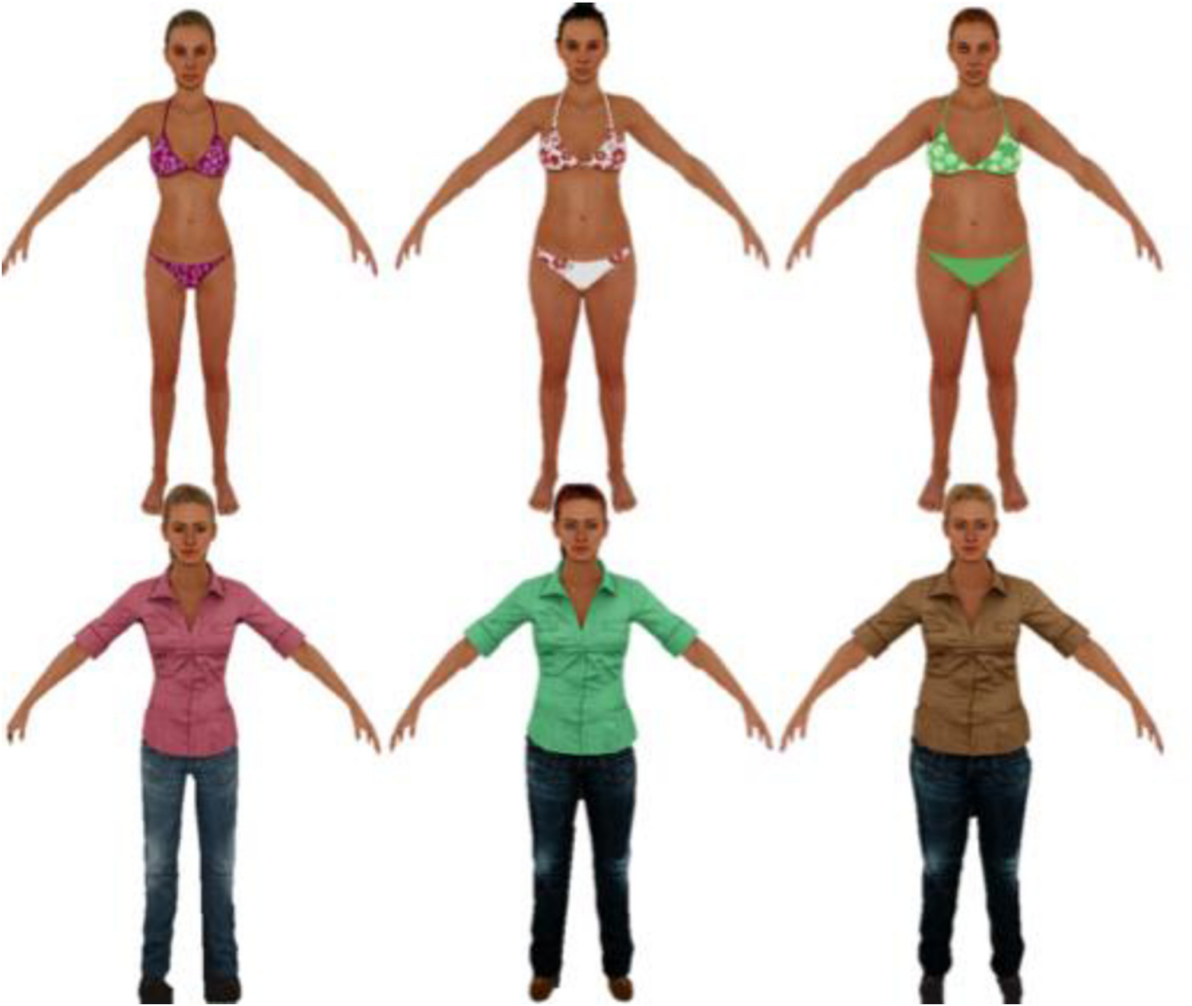
Female avatars from beach and party environments representing thin (BMI = 18), average (BMI = 23) and overweight (BMI = 28) from left to right.

The environments for the VR assessment task were designed to elicit varying levels of state BD. We used a 2x2 design (high vs. low) to vary the degree of (a) body salience and (b) social presence, resulting in four distinct environments. These factors were chosen based on previous research on state body dissatisfaction (state BD) in nonclinical samples using both traditional methods and VR exposure [26,27]. Body salience was conceptualized as the level of suggested exposure of one’s own body, and when applicable, the degree of visibility of others’ bodies. A virtual beach was created as the high body salience environment, and an indoor scene reminiscent of a university building was created for low body salience conditions (Fig 1). Social presence was indicated by the presence or absence of digital humans, called avatars. In the beach environments, participants were told to imagine that they were dressed in a bathing suit, and when present, avatars were dressed in revealing bathing suits. In the indoor scenes, no specific instructions were given about the participant’s appearance, and when avatars were present they were dressed in long-sleeved shirts and long pants.

In order to confirm that equipment was functioning properly, all participants were first exposed to a virtual training environment designed to mirror the physical room in which the study took place. In the training room, the participants practiced walking while wearing the equipment to gain familiarity with the experience of being immersed in the virtual environment.

After the initial training room, the participants were randomly exposed to the four virtual experimental conditions to control for order effects. All participants were exposed to an empty beach, a beach populated with avatars, an empty party scene, and a party scene populated with avatars. Populated environments contained three groups of three avatars, each composed of two female and one male avatar.

The female avatars varied according to body size between the three groups: underweight, healthy weight, and overweight body sizes were represented (the male avatar remained the same). The groups were designed to represent a BMI of approximately 18 (underweight), 23 (healthy weight), and 27.5 (overweight) respectively. The groups stood in fixed positions within virtual space, equidistant from the participant’s starting position. The placement of each group relative to the participant’s starting position was counterbalanced within and between participants to control for environmental and individual factors that may influence approach behavior.

In the environments containing avatars, participants viewed all three groups from their starting position and then approached (“joined”) each group in the order of their preference. Participants spent 15–20 seconds observing each group. In environments without avatars, participants approached three areas of interest in the environment and observed each area for 15–20 seconds.

In the interval between exposure to each experimental environment, participants completed a measure of state BD, by reading the items on a virtual screen projected in the virtual reality headset and speaking aloud the number on the scale corresponding to their answer for each item. The research assistant was blinded to the scale items and audio-recorded participants’ responses. Responses were also hand-recorded by the researcher. Immediately following exposure to the final environment and completing the final measurement of state BD, the research assistant removed the virtual reality headset and the participant completed post-test measures.

#### Apparatus

**Fig 2:**
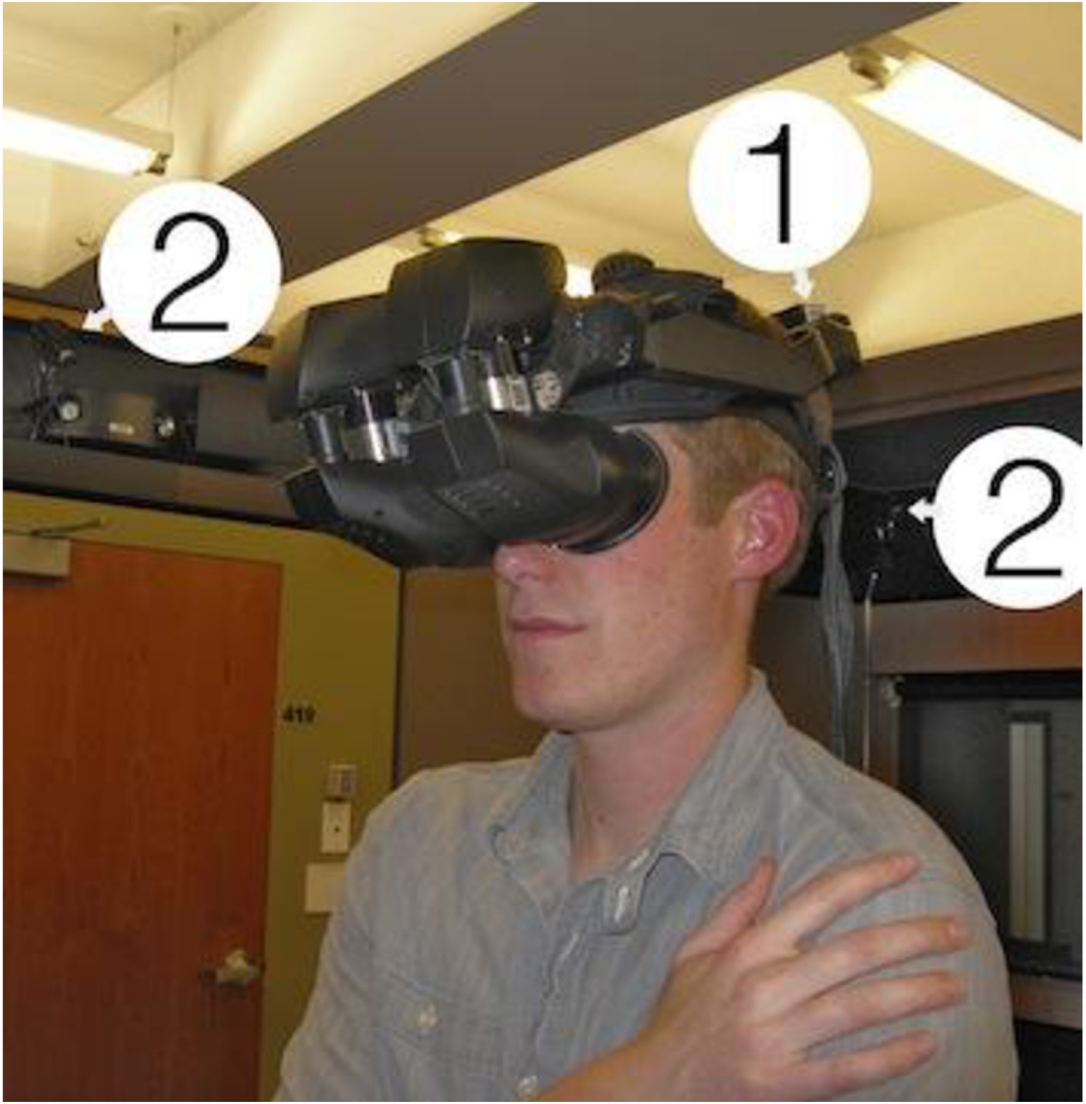
Subjects viewed the virtual environments using a head-mounted display (HMD). (1) An accelerometer allowed for naturalistic head movements during virtual exposure and (2) an optical infrared camera system recorded subjects’ gross body position in virtual space, allowing subjects to navigate the virtual environments via natural walking patterns.

Participants viewed the virtual environments using a head-mounted display (HMD), a virtual reality headset that provided three-dimensional stereoscopic views. They wore a nVisor SX111 HMD (NVIS, Reston, VA) with a resolution of 2560 x 1024 and a refresh rate of 120 frames per second (60 seconds per eye), enabling them to have naturalistic head movements while navigating the virtual worlds. An Intersense3 Cube accelerometer tracked their physical head movements (pitch, roll, and yaw) operating at 180 hertz with a 4-millisecond latency rate. In addition, participant’s gross body position on x-, y-, z-axis was tracked using an optical infrared camera system (Worldviz PPT-H) operating at 180 hertz with a 20-millisecond latency rate (with a precision of 0.25 millimeters). The optical tracking system allowed participants to navigate the virtual environment by walking naturally. Fig 2 illustrates the HMD, accelerometer, and optical infrared camera system. In addition, the physical lab room generated spatialized sound using a 24-channel ambisonic auralizer sound system (i.e. the location and direction of the sound in the IVE was reflected in the same location and direction as the physical lab space). All virtual environments were generated and programmed using Worldviz’s Vizard VR toolkit.

### Measures: Pre-VR

#### Weight-and-shape concerns

Researchers assessed weight-and-shape concerns using the five-item Weight Concerns Scale (WCS) [28]. This instrument assesses worry about weight and shape, fear of weight gain, dieting behavior, relative importance of weight, and frequency of feelings of fatness. The psychometric properties of the WCS are acceptable and the measure has been widely used in studies of eating behavior and BID [29,30].

#### Body mass index

Self-reported height and weight was recorded at baseline to calculate approximate BMI [BMI = weight(kg)/height(m)^2].

#### Eating attitudes and behaviors

The Eating Disorder Examination-Questionnaire (EDE-Q), a 39-item self-report version of the Eating Disorder Examination (EDE), is a well-established investigator-based interview used to diagnose and assess eating pathology [31]. The EDE-Q assesses eating disorder pathology over the past 28 days and provides a global score as well as four subscale scores (restraint, eating concerns, weight concerns, and shape concerns; [32]). The EDE-Q has shown acceptable reliability and validity as compared to the EDE interview among young adult women [33,34]. The EDE-Q was used to assess eating pathology at baseline, prior to VR exposure. Women with current EDs were not included in the present study for several reason: (a) BID is often severe among ED patients, and as such might not be expected to fluctuate according to environmental conditions in a manner comparable to non-ED women; (b) exposure to the environments could be triggering for women with ED; (c) this study aimed to explore BID among women with elevated weight-and-shape concerns but without current diagnosis of ED, as this is an important but understudied population in the BID literature.

### Measures: During VR

#### State body dissatisfaction (State BD)

State BD was measured using the Body Parts Satisfaction Scale–Revised (BPSS-R) [35]. The BPSS-R is a 10-item self-report measure that uses a Likert-type rating scale to assess the participant’s current level of satisfaction with ten body parts, resulting in a global score and two subscale scores (Satisfaction with Body scale and Satisfaction with Face scale) [35]. The measure has been shown to have acceptable psychometric properties among ethnically diverse undergraduate women [31]. The original measure was created to measure body satisfaction over the past month but it is also appropriate as an immediate measure. For the purposes of this study, only the Satisfaction with Body scale was analyzed.

#### Interpersonal distance from avatars

**Fig 3:**
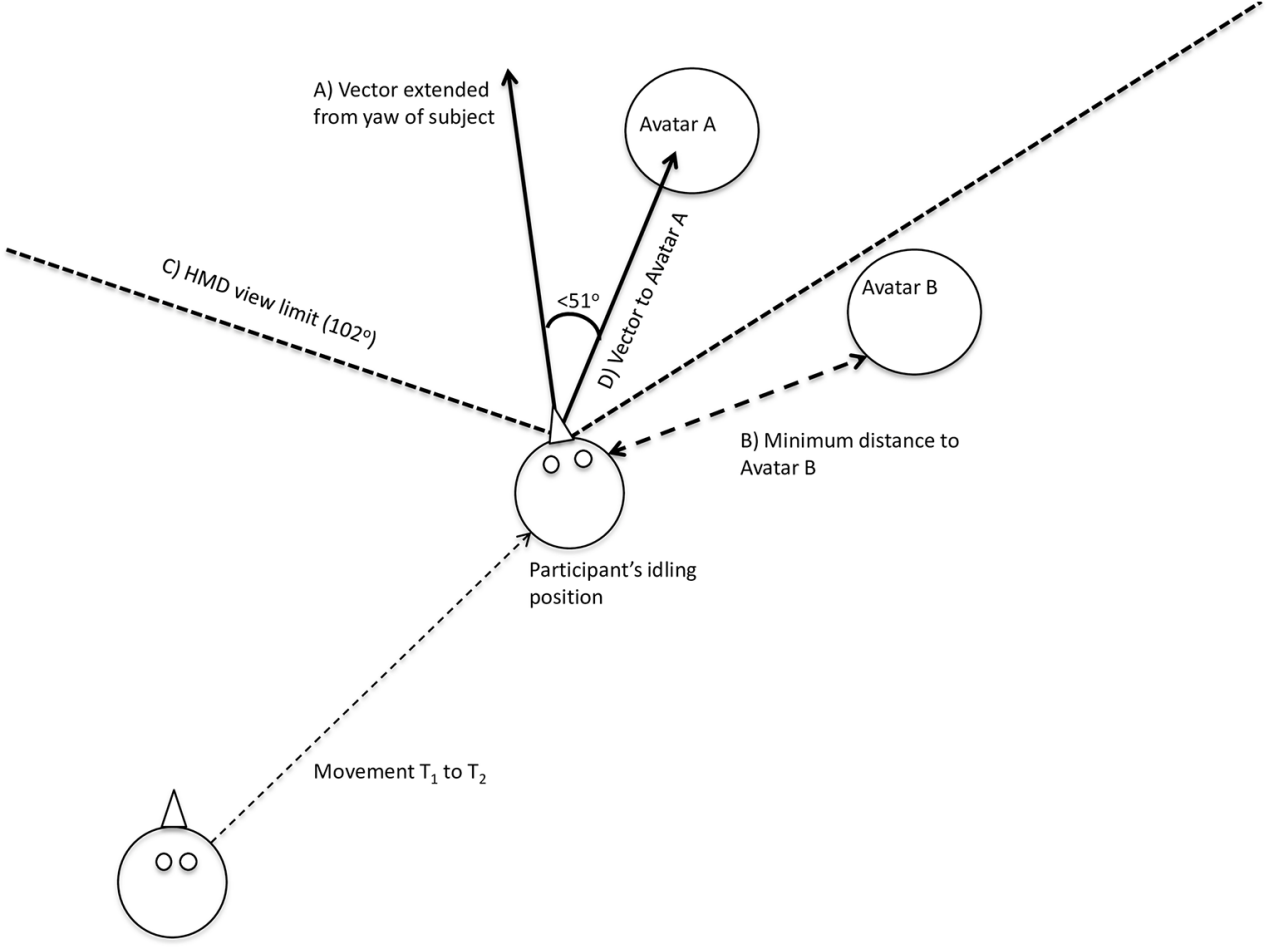
Aerial depiction of the method for calculating visual gaze and interpersonal distance. The participant’s movement from her starting point at T_1_ to her idling position at T_2_ is recorded by the optical tracking system in the laboratory room. At T_2_, line C indicates the participant’s entire horizontal field of view of 102^0^ within the HMD. Avatar A is within the participant’s range of view, whereas Avatar B is not in view. A vector (line A) was extended from the center of the participant's head along the z-axis (i.e., extending out from the nose), and another vector (line D) was drawn between the participant and Avatar A, providing a measurement for angle E. The participant is considered to be looking at a given avatar if angle E is less than half of her entire field of view, or less than 51^0^. Line B demonstrates the distance between the participant at T_2_ and Avatar B, measured as the distance from the participant to the outside edge of a cylinder with a radius equal to the width of Avatar B, as measured in Inspector.

Throughout virtual exposure, the VR tracking system collected data on participants x, y and z positions in the virtual environment at a rate of 60 frames per second, measuring participant’s position relative to the virtual objects in meters. A participant’s “minimum distance” was operationalized as the smallest absolute distance reached between a participant and each target avatar group. When calculating the distance between the participant and an avatar, analysis controlled for the relative body size of the avatar. First, the width of the avatar was calculated using the “Inspector” program of Vizard (WorldViz, Santa Barbara, 2012). In the analysis, the avatar was then considered to be a cylinder with a radius equal to the avatar’s calculated width, and the minimum absolute interpersonal distance was considered to be the smallest distance from the center of the participant to the outside edge of this cylinder, as shown in Fig 3.

#### Visual gaze toward avatars

Visual gaze was measured as the total percentage of time within each populated virtual environment that the participant spent looking at each avatar group. Participants’ head movement (yaw, rotation around the y-axis) was used to determine if they were looking at a group of avatars at a given time point (60 frames per second). Head direction, as opposed to eye movement, was used to indicate visual gaze. The technical challenges of measuring eye movement in immersive VR are significant, however head direction is very rarely decoupled from eye movement during VR exposure, and head direction is a more socially meaningful indicator of gaze than eye movement [20]. As shown in Fig 3, for each avatar in the group, a vector was drawn from the participant to the avatar. Next, the angle was calculated between the two vectors. If this angle was less than 51 degrees (half the view of the HMD worn by the participant), then the avatar was considered to be within the view of the participant. If at least one avatar was within the view of the participant, the group was considered to be in view. The participant was considered to be looking at a group only if it was the only group within her view at that frame according to this method. All time points at which avatars from more than one group were within the participant’s field of view were eliminated from analysis. We therefore calculated the total time in VR during which a participant was viewing one avatar group exclusively. All sensory cues in VR were held constant except for the position of the avatar groups, which was counterbalanced within- and between-subjects. This allows us to reasonably deduce that systematic differences in visual attention toward any of the groups was indeed the result of participants looking at the avatars, and was not influenced by other sounds or visual stimuli in VR.

### Approach Preference

We determined which avatar group each subject chose to approach first in each populated environment by calculating the direction of the subject’s initial movement in each environment relative to the positions of each avatar group. This was also hand recorded by the researcher while observing the participant’s movement (i.e., the researcher noted which group the participant chose to join first).

## Results

All data analyses were performed in R [36], and all mixed models were performed using lmerTest [37] in order to obtain *p*-values. Data was analyzed from participants who completed all assessments and completed all virtual exposures (*N* = 77). Linear mixed-effects models were used to examine the relationships between BID risk (i.e. Control, At-risk) and all relevant outcome variables (Appendices A-C). All models contained participants as random intercepts to account for within-subject correlations between repeated measures from the same person [38]. ANOVAs were used to compare models by testing the goodness of fit of the model to the data using the Maximum Likelihood method [38]. The less complex model was selected when there was no significant difference between the models in how well they fit the data. Contrasts used the average body size avatars and the empty beach scene as the comparison groups for the avatar body size and the virtual environment variables respectively. Statistical tests were two-tailed to allow for the detection of both positive and negative changes on target variables. A significance level of 0.05 was used for all analyses unless otherwise stated.

### Participant Characteristics

Table 1 presents the means and standard deviations for demographic and clinical characteristics for the sample. A general linear model compared the differences in baseline WCS by risk group, controlling for BMI, age, and race/ethnicity. There was a significant effect for WCS such that the At-risk group reported significantly higher levels of weight-shape concerns at baseline with no significant differences in BMI, age, or race/ethnicity: *R* ^2^ = 0.71, *F*(4, 71) = 42.85, p<0.001. WCS scores ranged from 5.0 to 95.0.

**Table 1:** Participant demographic and clinical characteristics.

	Control (*n* = 40)	At-risk (*n* = 37)
Age in years, *M* (*SD*)^**1**^	20.07(1.47)	20.67(2.52)
Race/Ethnicity (%)		
White/European Descent	50.00	48.64
African-American	10.00	2.70
Asian-American/ Pacific Islander	12.50	18.92
Latina/Hispanic	12.50	13.51
Middle Eastern-American	2.50	0.00
Other/ Prefer not to say	12.50	16.22
BMI, *M* (*SD*)	22.19(2.45)	22.70(1.96)
Weight Concerns Scale Score, *M* (*SD*)	24.77 (12.09)	61.70 (12.57)

^1^ Due to missing data for one participant, age analysis is based on *N* = 76.

### Body Satisfaction

Linear mixed-effects models were used to examine the association between BPSS-R scores, BID risk group, and virtual environment (S1 File- Appendix). Participants were modeled as random intercepts. BID risk, virtual environment, and BPSS-R scores measured after each virtual environment acted as fixed factors. The results from showed a significant difference in mean baseline BPSS-scores, with women in the Control group reporting higher body satisfaction compared to the At-risk group, *b* = 1.00, *t* = 27.4, 12, *p*<0.001. After controlling for baseline body satisfaction, there was no main effect of risk group on body satisfaction, *b* = 0.07, *t* = 0.93, *p* = 0.35.

In examining the virtual scenes, there was a significant effect of environmental condition regardless of risk. Participants reported less body satisfaction in the populated beach scene compared to the empty beach scene (S1 File- Appendix). Contrasts were applied to the model to further examine differences between the virtual environments. There were no differences in mean BPSS-R scores in the empty environments compared to the populated environments, *b* = 0.02, *t* = 1.49, *p* = 0.14, and no differences in mean BPSS-R scores in the beach environments (i.e. populated and empty) compared to the party environments (i.e. populated and empty), *b* = -0.02, *t* = -1.38, *p* = 0.17. However, there was a significant difference In BPSS-R scores between the populated beach environment compared to all other virtual environments, *b* = -0.02, *t* = -2.15, *p* = 0.03. Participants reported feeling the lowest level of body satisfaction in the populated beach scene when compared to all other virtual scenes.

**Fig 4:**
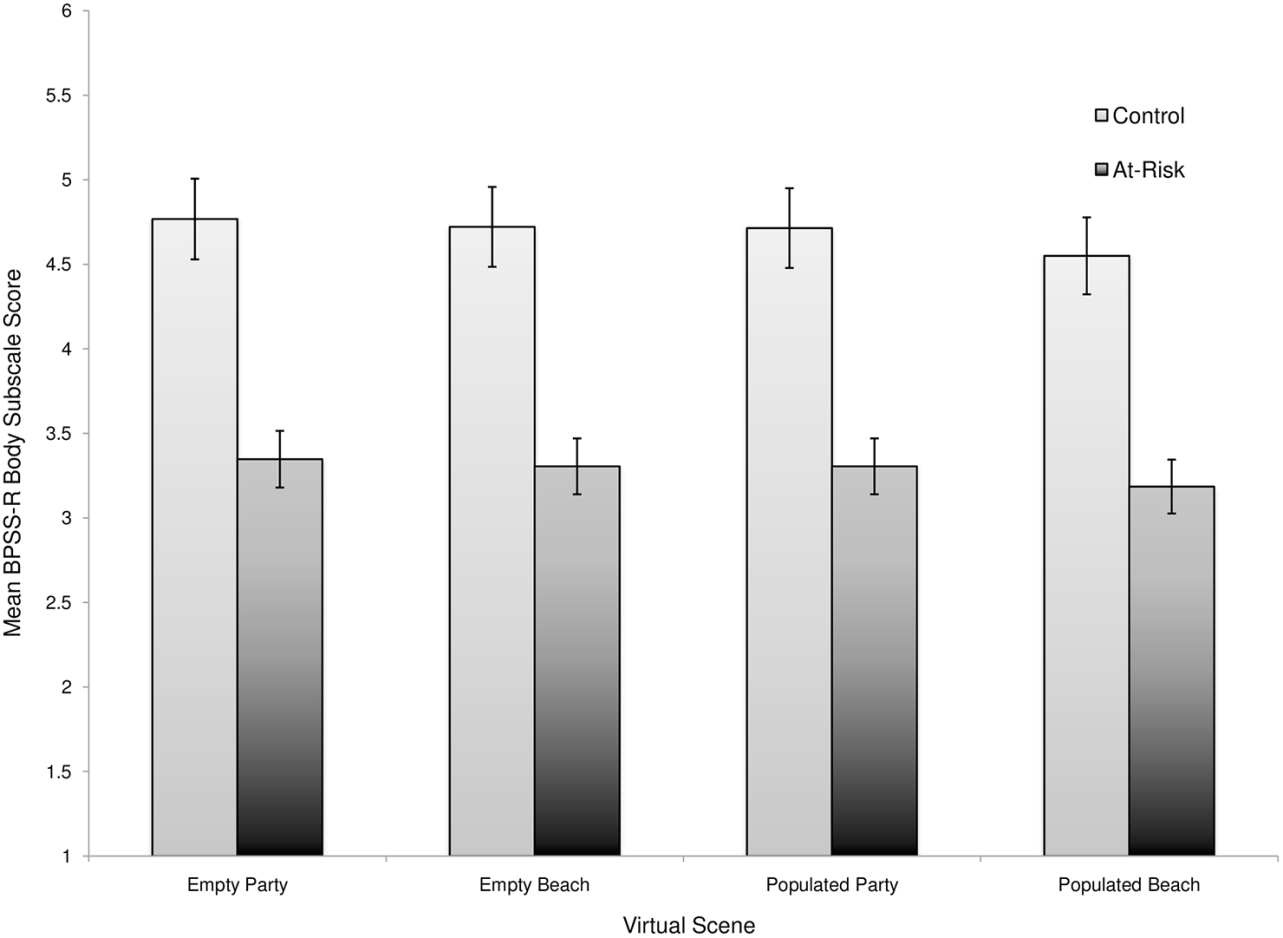
Mean BPSS-R Body Subscale scores for women with and without elevated weight-and-shape concerns. Error bars represent 95% confidence intervals.

Mean BPSS-R Body Subscale scores for both groups across all environments are presented in Fig 4.

### Interpersonal Distance

Linear mixed effects models were used to compare differences in participants’ minimum interpersonal distance from the avatars of different body sizes (average, overweight, and thin) in the two populated virtual environments (beach and party; S2 File—Appendix). Table 2 presents the means and confidence intervals for minimum interpersonal distance from avatars of each body size for at-risk and control group participants.

**Table 2:** Means and 95% confidence intervals for absolute minimum interpersonal distance from avatars of each body size for participants with elevated weight concerns versus control groups. Distances are reported in meters.

	Average Weight	Thin	Overweight
Control	0.54 (0.51–0.58)	0.57 (0.53–0.60)	0.60 (0.55–0.65)
At-Risk	0.59 (0.55–0.64)	0.57 (0.52–0.61)	0.59 (0.54–0.63)

There was no significant main effects of BID risk group, *b* = -0.05, *t* = -1.35, *p* = 0.18, or of virtual environment, *b* = -0.02, *t* = -1.74, *p* = 0.08, on minimum distance. There was a significant risk group and avatar body size interaction. The Control group minimum distance to the overweight avatars compared to the average sized avatars differed significantly from the At-Risk group’s comparison, *b* = 0.07, *t* = 2.29, *p* = 0.03. There were no differences between the average and thin sized avatar, *b* = 0.05, *t* = 1.79, *p* = 0.07 when compared to the At-risk group. There was no main effect of significant in minimum differences in how close participants stood to the overweight avatars compared to average sized avatars, *b* = -0.01, *t* = -0.28, *p* = 0.78, nor the thin avatars compared to the average sized avatars, *b* = -0.03, *t* = -1.19, *p* = 0.23.

Contrasts applied to the model further examine the interaction effects between BID risk group and avatar body size. The first contrast compared the interpersonal distance of the average-sized avatars against both the overweight and thin sized avatars. The second contrast compared the overweight sized avatars with the thin sized avatar. There was no main effect of avatar size comparing the overweight and thin sized avatars to the average body size group, *b* = -0.01, *t* = -0.86, *p* = 0.39. In addition, there was no differences in their minimum distance to the overweight avatar group compared to the thin avatar group, *b* = 0.01, *t* = 0.91, *p* = 0.36.

There was an interaction effect between risk group and these contrasts. The Control group demonstrated differences in minimum differences according to avatar body size compared to the At-Risk group. The Control group stood significantly closer to the average sized avatars compared to both the overweight and thin sized avatars, *b* = 0.02, *t* = 2.35, *p* = 0.02 in comparison to the At-risk group. The second contrast revealed that the Control group demonstrated no differences in the minimum distance between the overweight group compared to the thin sized group, *b* = 0.01, *t* = 0.50, *p* = 0.62 in comparison to the At-Risk group.

### Visual Gaze

Linear mixed effects models were used to examine the effect of avatar body size and virtual scene on the percentage of time participants spent looking at the avatar groups (S3 File—Appendix). There was no difference between the beach virtual environment and the party virtual environment, *b* = -0.05, *t* = -0.10, *p* = 0.91. There was a main effect of avatar size, with the thin avatars having a greater percentage of time being viewed compared to the average, *b* = 1.32, *t* = 3.04, *p* = 0.01. There was no difference in the percentage of time between the average sized avatar and the overweight avatar, *b* = -0.76, *p* = 0.32. However there was an interaction effect of BID risk group and avatar body size. The At-risk group spent more time looking at the thin avatars compared to the average-sized avatars, whereas Control group spent more time looking at average-sized as compared to thin avatars, *b* = 3.71, *t* = -3.46, *p* = 0.001 (see Table 3). There were no differences in how the Control group looked at the average sized avatars compared to the overweight group compared to the At-Risk group, *b* = -0.31, *t* = -0.29, *p* = 0.77. The Control group overall spent more timing looking at all the avatars compared to the At-risk group. This main effect of BID risk group approached, but did not reach, the level of statistical significance, *b* = 1.48, *t* = 1.96, *p* = 0.05.

**Table 3:** Means and 95% confidence intervals for the total percent of time within populated environments participants spent looking at avatar dyads of each body size.

	Average Weight	Thin	Overweight
Control	27.52 (26.30–28.75)	26.16 (25.17–27.15)	26.45 (25.23–27.67)
At-Risk	26.04 (25.18–26.90)	28.39 (27.26–29.51)	25.28 (24.32–26.23)

Using the average sized avatars as the comparison group, contrasts revealed additional interaction effects. The first contrast compared the average sized group against both the overweight and thin groups. The second contrasts compared the overweight avatar group with the thin sized avatar group. There was no significant difference in the amount of time that participants looked at the average sized avatars compared to both the overweight and thin avatars, *b* = 0.26, *t* = 1.17, *p* = 0.24. When comparing the overweight and thin avatars to each other, participants spent significantly higher percentage of time looking at the thin avatars compared to the overweight avatars, *b* = -1.55, *t* = -4.00, *p*<0.001. The Control group spent a significantly higher percentage looking at the average avatars compared to both the thin and overweight avatars, *b* = -0.67, *t* = -2.15, *p* = 0.03 in comparison with the At-Risk group. Finally, when only comparing the overweight avatars and the thin sized avatars, the Control group spent a significantly greater percentage of timing looking at the overweight avatars compared to the thin avatars, *b* = 1.70, *t* = 3.15, *p* = 0.002 compared to the At-Risk group.

### Approach Preference

**Fig 5:**
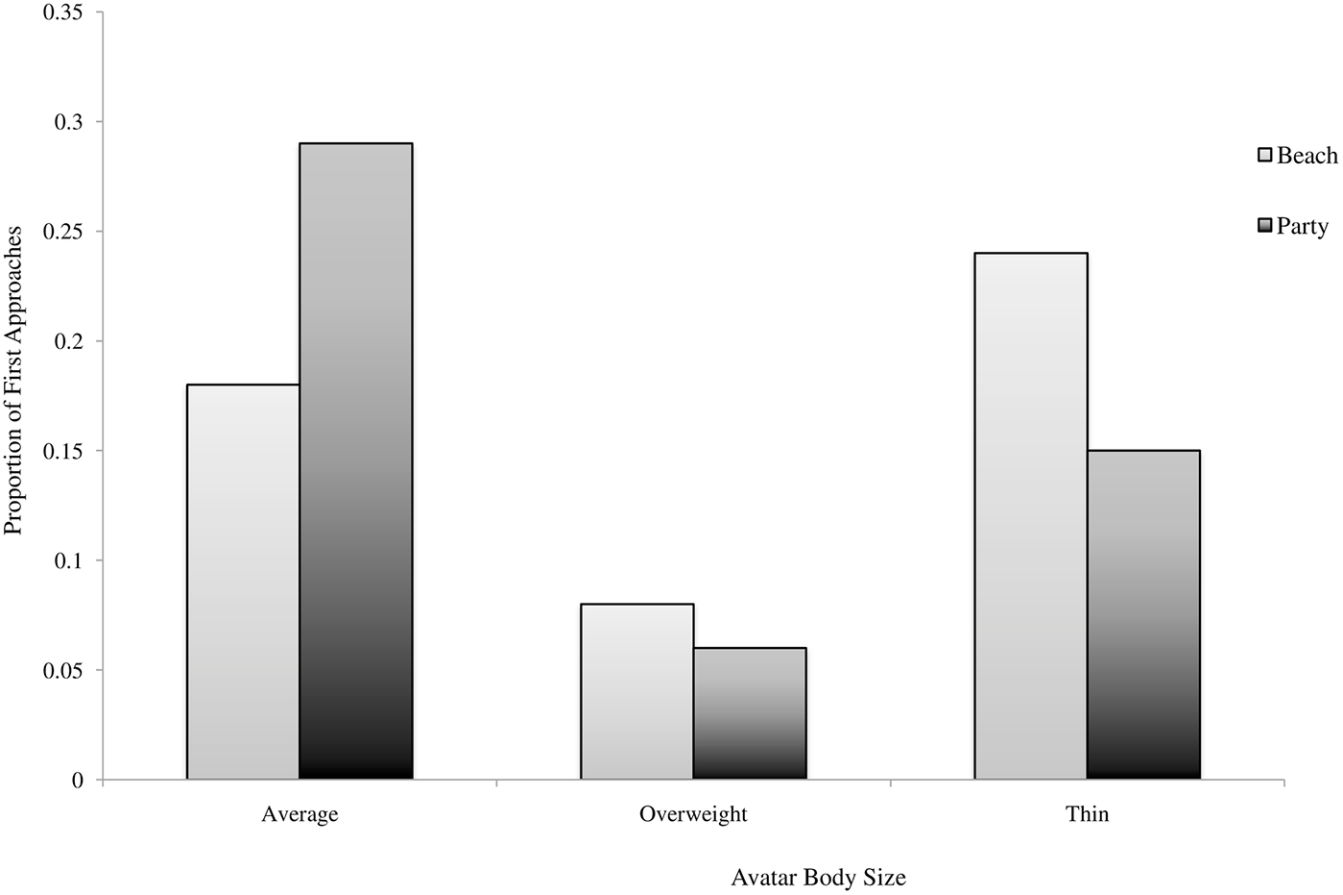
Proportion of avatars approached first by body size and populated environment.

Two chi-square test of goodness-of-fit tests, were performed to determine whether the three avatar groups were equally preferred in terms of approach behavior in the two populated virtual environments. Preference for the groups was not equally distributed in the beach environment, *x* ^2^ (2, *N* = 77) = 11.342, *p* = .003, Cramer’s *V* = 0.27, or in the party environment, *x* ^2^ (2, *N* = 77) = 25.66, *p* < .001, Cramer’s *V* = 0.41. As shown in Fig 5, regardless of their level of weight concern, participants were less likely to initially approach overweight avatars as compared to normal weight or thin avatars in both environments.

## Discussion

The present study investigated the use of VR in the study and assessment of BID. The primary aims of this study were (a) to identify environmental factors that relate to BID among women without eating pathology; and (b) to identify automatic behavioral indicators of BID. We found that all participants reported relatively stable state BD across a series of virtual environments, but greater state BD in a social environment with high body salience. With regard to behavior, we found that, compared to women without elevated weight and shape concerns, participants weight-and-shape concerns stood further from avatars of average weight and spent more time looking at thin female avatars than average or overweight female avatars. Finally, we found that all participants were reluctant to approach overweight female avatars.

Across all virtual environments, women with higher baseline weight-and-shape concerns reported lower body satisfaction than women in the control group. This was expected, and builds upon prior VR studies, which have shown that ED patients experience distress during exposure to virtual foods and eating environments [22]. We found that regardless of baseline weight-and-shape concerns, women reported slightly worse body satisfaction on the populated virtual beach. The environmental factors we manipulated in this study, body salience and social presence, had some influence on BID, although the effect was small. Interestingly, we found that state BD shifted across environments at a similar rate among women with and without pre-existing weight-and-shape concerns, suggesting that body salience in a social context may be have widely-applicable relevance for women’s body image concerns. Researchers interested in VR measurement of BID in sub-clinical ED populations might consider adopting a similar paradigm.

Contrary to our hypotheses, we did not find that interpersonal distance or approach preference toward thin avatars differed depending on BID. Given that BID is characterized by thin-idealization, it is surprising that we failed to find between-group differences with regard to behavior toward a thin avatar. Previous VR research using personal space as an outcome measure in human-avatar interaction has generally concluded that smaller interpersonal distances reflect greater affiliation or sense of “intimacy” between the subject and the target avatar [39]. However, it is important to note that higher-than-average thin-idealization is extremely common among college-age women[40,41]. Thus, it is possible that both groups held similar levels of thin-idealization, consistent with cultural norms, but women with BID lack a comparable affinity for healthy or “average” looking bodies, and instead hold a narrower and more extreme definition of “thin-ideal.” This could help to explain why, despite widespread cultural acceptance of the thin-ideal, not all women develop BID or struggle with disordered eating. Along these lines, we found participants with BID stood further from normal weight avatars than did participants without BID. It may be that more closely approaching normal weight avatars over other body types reflects an implicit preference for “healthy” body-cues among women in the control group. Body-dissatisfied women, on the other hand, might lack this “preference for healthy,” reinforcing maladaptive social cognitions. These findings suggest that research into protective mechanisms that may function to promote healthy body image may be as elucidating as studying pathological processes.

We further found that compared to the control group women with BID spent a greater percentage of time looking at thin avatars as compared to the other avatar groups. This is consistent with existing research findings on automatic attentional biases among women with BID, which has shown that women with BID demonstrate automatic attention toward thin female bodies. It is argued that such biases in attention and processing increase women’s feelings of body dissatisfaction [15,17,18]. That is, body-dissatisfied women tend to notice and attend to thin bodies more than other women do, thereby reinforcing the notion that “thin is in.” In contrast, when presented with avatars with a variety of body types, women in the control group devoted the greatest attention to normal weight avatars. It may be the case that this pattern of automatic attention serves a protective function for these women. Moreover, these findings suggest that the immersive quality and affective realism of VR may provide added benefit to existing attention re-training treatment approaches for BID.

Unexpectedly, we found that participants in the present study, regardless of condition, were reluctant to approach overweight avatars. Given that overweight avatars were identical to thin and normal weight avatars on all features except weight, it is reasonable to interpret this preference as largely driven by body size. Overweight and obese individuals are frequently the targets of social stigma and, particularly among adolescents, overt social exclusion [42,43]. The detrimental impact of weight stigma on the mental and physical wellbeing of overweight and obese individuals has been well documented [44,45]. Researchers have largely relied upon the Implicit Association Test as a primary outcome in studies aimed at assessing and/or modifying weight stigma. We are aware of only one study to investigate behavioral measure of weight stigma: Bessenoff and Sherman [46] used interpersonal space in a real world waiting room paradigm to measure participants’ willingness to approach an obese versus normal weight target. They found that participants with implicit anti-fat attitudes sat further away from a chair believed to be occupied by an obese woman.

The results of the present study suggest that immersive virtual environments may offer unique advantages to measure weight stigma at the behavioral level. Such methods may inform the development of more robust interventions to reduce stigmatized beliefs about overweight and obese people, which prove difficult to modify through more traditional, dissonance-based approaches [43,47]. VR has been used to study social behaviors toward other stigmatized groups; for example, researchers have measured interpersonal distance as an indicator of participants’ level of stigmatization toward avatars who are perceived to be HIV-positive [48,49]. Further, one study recently investigated the use of virtual embodiment to reduce self-criticism in a non-clinical sample of young women, suggesting that VR may show promise as a tool to reduce the detrimental effects of self-stigmatization among members of marginalized groups, including people with obesity [50].

The present study may have important implications for the development of BID treatments. For example, the current set of environments could be expanded to provide an array of salient practice situations, as has been done in virtual exposure-based treatment for specific phobias and PTSD [51,52]. For instance, virtual environments could be used in exposure-based treatments; patients with high BID could practice behavioral skills in virtual environments and then apply them to real environments. The VR paradigm developed in this study was completed in less than 30 minutes, which may make it palatable to clinicians for whom time considerations are essential. There is promising evidence to suggest that implicit interventions aimed at correcting maladaptive automatic processes may be effective at improving BID [14,53,54]. The present VR task could be used to expand on existing implicit interventions by incorporating overt behavior and naturalistic settings.

Virtual spaces are increasingly common components of modern social life. Millions of people interact in virtual social spaces each day, some of which involve embodiment with an avatar such as online role-playing games [55]. As novel forms of social interaction continue to evolve, it is important for psychological research to progress in tandem. The present study may contribute to a preliminary understanding of BID as it relates to virtual social interactions. In particular, our findings suggest that as users navigate virtual social spaces, preexisting body image concerns remain salient, even when individuals have no direct feedback regarding their own virtual appearance. As this field of research develops, it will be important to consider how virtual interactions and avatar embodiment, among other factors, may influence BID within a virtual setting. Developing more nuanced understanding of BID in the context of virtual spaces, incorporating such themes as embodiment, interaction with avatars, and one’s self-perspective in VR, may allow for the development of universal, disseminable virtual interventions to improve body satisfaction.

### Limitations

First, our relatively homogenous sample may be a limitation of this study. Because participants were recruited only from a small number of courses within the Communications Department, the sample may not be representative of college women in general. The final sample had a high percentage of white participants, and a relatively small range in age and BMI, which may reduce the generalizability of these findings. For example, our sample contained very few women with BMI > 25, which may limit our ability to draw inferences about the influence of an individual’s weight status on behavior, state BD, or their approach behaviors toward overweight avatars. In addition the present study did not include direct measurement of participants’ stigmatizing beliefs or attitudes, which limits our ability to interpret participants’ avoidance of overweight avatars. Future studies should recruit higher weight participants in order to better understand how personal characteristics may influence approach behavior, and more comprehensive studies are needed in order to draw strong conclusions. Finally, while men are certainly affected by BID, research on male experiences of body image is limited. Existing research indicates that BID is expressed differently in men than in women, making it difficult to extend existing models of BID to a male population. Moreover, there is no research on attention biases related to BID using male participants. In order to allow us to draw clearer conclusions based on prior work with women, we did not include male participants in the present study. Future research extending models of BID to apply to men as well as women is clearly indicated.

## Conclusions

The current results suggest that VR provides effective tools for measuring BID in realistic settings. The present study provides preliminary evidence for environmental factors that are relevant for BID among non-ED women, although further research is needed to strengthen this paradigm. This is the first study to use VR to investigate implicit behavioral indicators of state BD, which may be helpful in measuring treatment outcomes or designing future behavioral exposure treatments. Emerging models increasingly indicate that automatic processes play an important role in the etiology and maintenance of BID, thus it is vital that researchers establish effective approaches by which to measure and understand such processes. The present study introduces a novel measurement of automatic behavioral processes associated with BID, lends support to growing evidence of visual attention biases in BID, and provides preliminary evidence that BID is also associated with specific patterns of automatic social behavior that may maintain the disorder.

## Supporting Information

S1 Dataset Complete dataset(s) for the present study. (ZIP)

S1 File Appendix. Table A. Linear Mixed-effects Models for Body Satisfaction by Virtual Scene. (DOCX)

S2 File Appendix. Table B. Linear Mixed-Effects Models for Interpersonal Distance by Risk, Avatar Body Size, and Scene. (DOCX)

S3 File Appendix. Table C. Linear Mixed Models for Visual Gaze by Risk, Avatar Body Size, and Scene. (DOCX)
