# Supplementary material for: Developing a Novel Measure of Body Satisfaction Using Virtual Reality
Source: PLoS One. 2015 Oct 15;10(10):e0140158. doi: 10.1371/journal.pone.0140158 (PMC4607468; doi:10.1371/journal.pone.0140158)
Supplement: S1 File — (DOCX) [file pone.0140158.s002.docx]

**Table A.** Linear Mixed-effects Models for Body Satisfaction by Virtual Scene

|  |  |  | *s^2^* | *b* | *SE* | *t* | *p* |
| --- | --- | --- | --- | --- | --- | --- | --- |
| Model: **Risk+Scene+Baseline** | |  |  |  |  |  |  |
| Random | Subject | Intercept | 0.04 |  |  |  |  |
| Fixed | *Risk* (Control)  *Scene* (Empty Party)  *Scene* (Populated Beach)  *Scene* Populated Party)  *Baseline* BPSS-R |  |  | 0.07  0.05  -0.14  -0.00  1.00 | 0.07  0.03  0.03  0.03  0.04 | 0.93  1.43  -4.36  -0.01  27.40 | 0.35  0.15  <0.001  1.00  <0.001 |

| Model with contrasts: Risk+Scene+Baseline | |  |  |  |  |  |  |
| --- | --- | --- | --- | --- | --- | --- | --- |
| Random | Subject | Intercept | 0.05 |  |  |  |  |
| Fixed | *Risk* (Control)  *Scene* (Empty vs. Populated)  *Scene* (Beach vs. Party)  *Scene* (Populated Beach vs. Other)  *Baseline* BPSS-R |  |  | 0.07  0.02  -0.02  -0.02  1.00 | 0.07  0.02  0.02  0.01  0.04 | 0.93  1.49  -1.38  -2.15  30.00 | 0.34  0.24  .17  0.03  <0.001 |
