# Supplementary material for: Developing a Novel Measure of Body Satisfaction Using Virtual Reality
Source: PLoS One. 2015 Oct 15;10(10):e0140158. doi: 10.1371/journal.pone.0140158 (PMC4607468; doi:10.1371/journal.pone.0140158)
Supplement: S2 File — (DOCX) [file pone.0140158.s003.docx]

**Table B**. Linear Mixed-Effects Models for Interpersonal Distance by Risk, Avatar Body Size, and Scene

|  |  |  | *s^2^* | *b* | *SE* | *t* | *p* |
| --- | --- | --- | --- | --- | --- | --- | --- |
| Model: Risk*Size+ Scene | |  |  |  |  |  |  |
| Random | *Subject* | Intercept | 0.02 |  |  |  |  |
| Fixed | *Risk* (Control)  *Size* (Overweight)  *Size* (Thin)  *Scene* (Party)  *Risk * Size* (Control: Overweight)  *Risk * Size* (Control: Thin) |  |  | -0.05  -0.01  -0.03  -0.02  0.07  0.05 | 0.04  0.02  0.02  0.01  0.03  0.03 | -1.35  -0.28  -1.19  -1.74  2.29  1.79 | 0.18  0.78  0.23  0.08  0.02  0.07 |
| Model with contrasts: Risk*Size+Scene | |  |  |  |  |  |  |
| Random | *Subject* | Intercept | 0.02 |  |  |  |  |
| Fixed | *Risk* (Control)  *Size* (Average vs. Thin/Overwt)  *Size* (Thin vs. Overwt)  *Scene* (Party)  *Risk*Size*  (Control: Average vs. Thin/Overweight)  *Risk*Size* (Control: Thin vs. Overwt) |  |  | -0.01  -0.01  0.01  -0.02  0.02  0.01 | 0.04  0.03  0.03  0.03  0.04  0.04 | -1.48  -0.65  -2.13  -1.99  1.63  1.86 | 0.14  0.52  0.03  0.05  0.10  0.06 |
