# Supplementary material for: Developing a Novel Measure of Body Satisfaction Using Virtual Reality
Source: PLoS One. 2015 Oct 15;10(10):e0140158. doi: 10.1371/journal.pone.0140158 (PMC4607468; doi:10.1371/journal.pone.0140158)
Supplement: S3 File — (DOCX) [file pone.0140158.s004.docx]

**Table C.** Linear Mixed Models for Visual Gaze by Risk, Avatar Body Size, and Scene

|  |  |  | *s^2^* | *b* | *SE* | *t* | *p* |
| --- | --- | --- | --- | --- | --- | --- | --- |
| **Model: Risk*Size + Scene** | |  |  |  |  |  |  |
| Random | *Subject* | Intercept | 0.00 |  |  |  |  |
|  | *Risk* (Control)  *Size* (Overweight)  *Size* (Thin)  *Scene* (Party)  *Risk * Size* (Control: Overweight)  *Risk * Size* (Control: Thin) |  |  | 1.48  -0.76  2.34  -0.05  -0.31  -3.71 | 0.76  0.77  0.77  0.44  1.07  1.07 | 1.96  -0.99  3.04  -0.11  -0.29  -3.46 | 0.05  0.32  0.003  0.91  0.77  0.001 |

| Model with contrasts: Risk*Size + Scene | |  | | |  | |  | |  | |  |  |
| --- | --- | --- | --- | --- | --- | --- | --- | --- | --- | --- | --- | --- |
| *Subject* | Intercept | | 0.00 |  | |  | |  | |  | |  |
| *Risk* (Control)  *Size* (Average vs. Thin/Overwt)  *Size* (Thin vs. Overwt)  *Scene* (Party)  *Risk * Size* (Control: Average vs. Thin/Overwt)  *Risk * Size* (Control: Thin vs. Overwt) |  | |  | 0.14  0.26  -1.55  -0.05  -0.67  1.70 | | 0.44  0.22  0.39  0.44  0.31  0.54 | | 0.32  1.17.  -4.00  -0.11  -2.15  3.15 | | 0.75  0.24  <0.001  0.91  0.03  0.001 | |  |
